# Implementing a research-based innovation to generate intra-familial involvement in type 2 diabetes self-management for use in diverse municipal settings: a qualitative study of barriers and facilitators

**DOI:** 10.1186/s12913-020-5036-7

**Published:** 2020-03-12

**Authors:** Tue Helms Andersen, Dan Grabowski

**Affiliations:** Steno diabetes Center Copenhagen, Neils Steensens Vej 2, 2820 Gentofte, Denmark

**Keywords:** Health care implementation, Family support, Patient education, Type 2 diabetes, Educator role

## Abstract

**Background:**

Involving family members in disease management is vital to people with type 2 diabetes. New innovations that support family members’ involvement can help both the person with type 2 diabetes and the relatives to create the supportive environment they need. The objective of the present study is to examine what facilitates and obstructs implementation of an innovation that supports intra-familial involvement in life with type 2 diabetes.

**Methods:**

Of the 48 healthcare professionals trained in facilitating the innovation in municipal patient education courses, single, semi-structured interviews were conducted with 13 of them. The interviews were focused specifically on the implementation process. All interviews were transcribed verbatim and analyzed using radical hermeneutics.

**Results:**

The analysis revealed three distinct themes affecting implementation of the innovation. 1) Focusing on creating family involvement in patient education for people with type 2 diabetes was relevant and important to the healthcare professionals. 2) The dynamics of group-based patient education sessions changed when family members were involved, which affects healthcare professionals’ group facilitation methods. 3) Implementing new methods in patient education requires great commitment and support from the organization and management.

**Conclusion:**

Implementation of an innovation to involve families and close relatives in patient education in Danish municipalities is feasible, but highly dependent on the commitment of healthcare professionals and managers as well as their openness to new ways of facilitating group processes.

## Introduction

Involvement of close relatives and family members in diabetes management is essential to people with type 2 diabetes [1, 2]. Nevertheless, intra-familial support is a complex matter involving different expectations and needs, and unvoiced concerns may lead to both supportive and obstructive behavior [3–5]. Furthermore, the close relatives of people with type 2 diabetes have a higher risk of developing type 2 diabetes themselves [6–8].

Familial challenges in life with type 2 diabetes can be categorized into six domains: knowledge, communication, support, everyday life, roles and worries [9]. Additionally, barriers to intra-familial prevention of type 2 diabetes can be divided into three areas: 1) Sole responsibilities and the absence of collective practices, 2) intra-familial differences in perception of risk and 3) lack of perceived disease significance [10]. These challenges and barriers support the notion that involving family members is highly complex, but also has great potential.

To understand the full potential of family involvement, there is a need for well-documented innovations and documentation of the implementation process [11]. A systematic review of reviews emphasized that there is a considerable body of literature on implementation strategies that focus on changing professional’s behavior or daily practice [12]. Nevertheless, identifying the most effective strategies and determining under what conditions they should be applied remain a challenge [12, 13]. In other words, there is a lack of clarity regarding terms, definitions and concepts [14].

Nilsen argues that implementation science often uses theories with three different objectives, which are to: 1) describe the process of translating research into practice, 2) examine the factors that influence implementation outcomes and 3) evaluate the effect of implementation strategies [15]. The present study focuses on the second objective.

There is, however, no one-size-fits-all systematic method for identifying the determinants of practice when designing the implementation. This calls for a mix of methods [16], and there is a need for more complex implementation strategies that address the type of knowledge involved, the context of implementation, the people involved, and the processes applied – all factors that are important to understanding the knowledge translation process [17]. This is, however, in contrast to an overview of systematic reviews which argues that single-component interventions are just as effective as multifaceted ones [18].

The present implementation study focuses on implementing tools to help healthcare professionals support people with type 2 diabetes and their families. When translating knowledge into practice, toolkits can work as a knowledge translation strategy to help facilitate the implementation of evidence. Toolkits have the potential to change healthcare practice, but to fully compare different strategies, future studies need to systematically describe both development and evaluation [19].

Successful implementation of tools in complex settings is highly dependent on the specific context. Consequently, implementation strategies must consider the changing context of the implementation settings [20, 21]. Scaccia et al. stressed that organizational readiness to implement new innovations involves: (a) the motivation to implement an innovation, (b) the general capacities of an organization, and (c) the innovation-specific capacities needed for a particular innovation [22]. Oborn et al. analyzed the gap between research and implementation using the concept of Knowledge Translation. They emphasized the importance of showing great openness to new innovations while at the same time maintaining every-day practice – all to narrow the knowledge gap in the settings targeted in the implementation process [23].

Cochrane et al. divided the barriers to implementation into the following categories: behavioral barriers, rational-emotive barriers, physician barriers, evidence barriers, patient barriers, resource barriers and process barriers. They stressed the need to examine the many complex factors that are essential for the knowledge-to-action process [24].

Furthermore, implementing self-management support in healthcare has been shown not to be well suited to a biomedical focus, causing it to become a lower priority for management, in turn resulting in less effort on the part of healthcare professionals [25]. The barriers identified may vary across settings and from one profession to another, but adapting the intervention to identified barriers is of great importance. Baker et al. showed that interventions that consider prospectively identified determinants of practice are more likely to be successful than no interventions and dissemination of non-adapted guidelines [26].

We need to know more about how to implement innovations that support healthcare professionals’ work with guiding chronically ill persons and their families. Based on the findings and arguments presented above, the objective of the current study is to examine the facilitators and barriers that affect implementation of an innovation to promote family involvement in type 2 diabetes patient education.

## Methods

### Prior development of family tools for patient education

In Denmark, patient education for people with type 2 diabetes is mainly carried out through the municipalities. It is often structured as weekly meetings for 6–10 weeks, where nurses, dieticians, physical therapists, etc., facilitate education on how to live with the disease. The topics include diet, physical activity, medicine and social support.

Only rarely are family members and other relatives invited to participate, and when they are, their role is often to observe rather than to be actively involved.

To change this, we developed a toolbox (The Family Toolbox) that is intended to support family members’ involvement in patient education. The toolbox was developed through a systematic design thinking process together with people with type 2 diabetes, their families and healthcare professionals. It contains four tools with distinct purposes and a plan for how to use all four in a patient education course adapted to people with type 2 diabetes and their families. The tools are: **a) The Family Mirror** gives people with type 2 diabetes and relatives a chance to create a vision of themselves and their view on life with type 2 diabetes in the family. It is used for self-reflection and to facilitate communication about feelings, needs and wishes within the family **b) The Family Book** offers an easy-to-read shared family book on various aspects of family life with type 2 diabetes. The focus is on knowledge that is of practical relevance to the families. **c) The Family Line** enables participants to reflect on, show each other and discuss how big a part of their life diabetes is. Furthermore, it allows participants to discuss how big a part diabetes should play in their lives and why. **d) The Family Plan** gives the families a chance to identify shared challenges and opportunities for action and to make a clear and solid plan for how to act.

The tools are based on solid knowledge from a comprehensive needs assessment involving people with type 2 diabetes, their families and healthcare professionals. The needs assessment identified six interconnected problem domains of significant importance to family members [9]. Additionally, the needs assessment provided us with three main barriers to intra-familial prevention in the families [10]. The six domains and three barriers were utilized when developing the four tools. Finally, an evaluation study based on interviews with 18 families after working with the Family Toolbox showed the value experienced by the families, which supports the importance of the present implementation study [27]. These three studies and the development of The Family Toolbox are all part of the same research project leading up to the present study of implementation.

### The present study: implementation process and competence development for healthcare professionals

The present study was based on interviews with healthcare professionals after implementing The Family Toolbox in complex municipal settings in Denmark. It focused solely on the implementation process.

To best support implementation of the Family Toolbox, we created a 6-week-long competence development course together with two of the five Danish regions: The Capital Region of Denmark and The Southern Region of Denmark. It consisted of two non-consecutive days of classroom training on how to effectively utilize the tools in patient education as well as background knowledge on family perspectives. Between the two classroom sessions, there were 6 weeks of testing in which the healthcare professionals were tasked with trying the tools in real-life settings and reflecting on as well as discussing the outcome.

Along with the Family Toolbox and the competence development course, we provided the healthcare professionals with a detailed guide to knowledge about family support and involvement, skills for facilitating family members and a hands-on guide to effective use of the tools.

### Interviews

For the present interview study, we had access to a population of 48 healthcare professionals who had finished the competence development course during the past 6 months. We used purposive sampling [28] to choose participants based on the following criteria: Region, Profession and Experience. We contacted the healthcare professionals by e-mail. During the recruitment process 7 healthcare professionals declined the invitation and 4 did not respond to our e-mail.

We performed semi-structured individual interviews with 13 healthcare professionals. The final sample (see Table 1) included six healthcare professionals from The Capital Region of Denmark and seven from The Southern Region of Denmark. Eight were nurses, three were dieticians and two were physical therapists. On average, the participants had 4.5 years of experience with patient education for people with type 2 diabetes.

**Table 1:** Participant characteristics

Int. no.	Region	Profession	Years of ex
1	South	Dietician	5
2	Capital	Nurse	6
3	Capital	Nurse	9
4	South	Nurse	3
5	South	Dietician	1
6	Capital	Nurse	3
7	Capital	Physical therapist	4
8	South	Nurse	5
9	Capital	Nurse	3
10	South	Physical therapist	11
11	Capital	Nurse	4
12	South	Nurse	2
13	South	Dietician	2

Interviewed healthcare professional by region, profession and years of experience

After 10 interviews we made an outline of overall themes in each of the individual interviews. This showed us that data saturation had not been achieved, as new themes had emerged in interview 9 and 10. We then did three more interviews and it was then established that no new overall themes had emerged since interview 11.

One researcher conducted all of the interviews. Focus themes in the interviews were: experience with patient education, experience with family involvement, facilitators of and barriers to working with the Family Toolbox.

### Setting

The interviews were conducted at the healthcare professionals’ respective workplaces. They took place during office hours and at a time suited to the healthcare professionals’ schedule, the goal being to make it as easy as possible to participate. The interviews were conducted in private rooms to ensure an open and honest conversation.

### Analysis

All interviews were transcribed verbatim and analyzed using radical hermeneutics, which are guidelines for content analysis that, by virtue of being a combination of hermeneutics and constructivism, manage to be both empirically true and theoretically complex [29]. Radical hermeneutics focuses on keeping a balance between theory, method and data, as an interconnected process that requires a constant focus on how these elements influence each other. The methodology entails three steps of analysis. The first step involves a reading of the data with a view to observing how specifically selected guiding differences are observed in the data. This observation in itself constitutes an interpretation rather than a description, and its purpose is to reduce the complexity of the data. Elements within the scope of the guiding differences selected by the interpreter are extracted from the data. The second step involves making these elements the subject of another interpretation, thus revealing new guiding differences. The third step involves interpreting the sum of these differences.

In the concrete analysis, the initial guiding differences focused on barriers to and facilitators of implementation. This first reading reduced the complexity of the data significantly. The second interpretation revealed the guiding differences, presented below as three themes. In the third round, these themes were analyzed and interpreted separately.

## Results

The analysis revealed three distinct themes. Each theme has facilitators and barriers that are embedded as important parameters of success.

1. The family as a potential focal point when educating people with type 2 diabetes

The healthcare professionals stated that focusing on family involvement in patient education has great potential. Thematically it immediately provided them with a set of reasons to carry out education that targets the whole family.

Most of the healthcare professionals had very little experience with involving family members as equal participants. For some of them, the theme of involving families in life with diabetes had not been part of the course:

*This is the first time I’ve done it (invite family members) now after the course. I had a few couples participating earlier, but only because they both had diabetes and wanted to join the same course. But I’ve never focused on families or relatives before. (Interview 2).*

While some had not even thought about family members, others had experience with debating the role of the family in patient education without them participating:

*I actually think there are many times during the course when we’ve included family and friends – their entire network – and talked about them a lot, but the relatives weren’t there so they didn’t hear it unless the people with diabetes went home and told them. (Interview 4).*

Having discussions about how to involve family and friends in daily life with diabetes was a common practice in patient education. Implementing what they learned during the discussions into daily life was otherwise difficult, and the healthcare professionals seemed to view family involvement as the missing link:

*The best thing was to start focusing on how important it is to involve relatives. I think it’s a long process to go from not involving relatives at all to doing it. So, I think it’s really positive to start involving the family in the patient education course 3–5 h a week. It’s at home that they’re going to make the changes, where many hours are spent, and here it’s precisely the relatives who can support them. If they’re not here and being supportive, I think it’s almost impossible to change your lifestyle. (Interview 4).*

The healthcare professionals often mentioned how people with diabetes have a hard time managing and maintaining lifestyle changes when they are at home and do not have support from the healthcare system. It is this crucial gap that families have the potential to fill.

Among healthcare professionals, there was a general and strong focus on the daily life of the people with diabetes participating in patient education. This appeared to be a strong driving force among the educators when they were asked why they wanted to participate in implementing the present innovation:

*It makes really good sense that they hear the same things and that this lifestyle change they are going to make is made rationally and with a common understanding. I can both see and hear that they are having totally different conversations after participating in a patient education course together. (Interview 2).*

Experiencing that the course was meaningful for both the people with diabetes and their families was of great importance to the implementation process. Most healthcare professionals participating in the present study brought this up as their primary reason for being eager to participate, and for why they wanted their colleagues and manager to participate as well.

2) The family focus creates a different role as facilitator in patient education

A common barrier to implementing the Family Toolbox was the fear of facing a larger and more diverse group of people when instructing and facilitating patient education courses. Splitting the large group into small groups that are working on how to live with diabetes was central to working with the Toolbox.

The risk of losing control during these group exercises played a key role for the healthcare professional:

*At first I thought, well, if I go out there and it turns into chaos, and it’s not good enough, and we come too close. What do I do then? So, I had thought about what to do if I had to wrap it up. I also felt a little nervous to see if I was skilled enough, but it went really well. No problems. (Interview 3).*

In the quote, the nurse felt the need to prepare for the worst, because of the risk of losing control in a new situation that involved dealing with families. As it turned out, the thorough preparation provided good opportunities to end the session successfully. Not everyone had the same experience:

*It was suggested that you could use it (The Family Toolbox) in a group with many families, and that one or two facilitators, depending on the number of families, could go around from family to family. I’ve only had one experience with it, but my experience is that the situation could be quite vulnerable, that there is no third part to bring balance into the families and make the ends meet. This is not to say that we have the solution, but in that couple, if we hadn’t been there, I think they would have ended up on the same old track. (Interview 8).*

This nurse did not believe that he/she would be able to support more families working with the tools at the same time. This might entail the families losing track of how to work with the tools and leaving without having moved beyond the same old discussions that lead nowhere. The healthcare professional cannot take that kind of risk. This constitutes a great barrier to implementation, and it would seem that one questionable experience with the tools could reduce a professional’s willingness to test the tools further.

Others reacted differently to facilitating, even though they also had their doubts:

*And here we had some discussions about whether we should sit at the table with them, or if they really should have this conversation themselves. I’m not sure about what’s best actually. In the last group I told them, I would be their assistant, and that they could call me if they thought the conversation was stuck or they wanted me to clarify a question or something. Then I let them start themselves. (Interview 13).*

This shows that while some of the healthcare professionals needed to control how each session moved forward, others were keener to explore new behaviors. The willingness to facilitate in new settings, with the risk of losing control of the session, gave successful implementation a greater chance.

Another important aspect of implementing successfully was preparing the healthcare professionals to work with the tools and the families. In the interviews, the healthcare professionals often referred to the competence development course when describing how they developed their use of the tools:

*I’ve thought about whether it was too wild to present it to them the first time, but I’m not afraid of that anymore. When I was taking the competence development course, I tried it during a day course with people with diabetes who’s met several times before, so they knew each other quite well. I gave them the Family Mirror and I asked them to present themselves and their life with diabetes, but also to present what they thought their relatives where thinking about diabetes. So they had to talk about themselves as well as their relatives in couples and it actually worked really well. (Interview 2).*

Trying out new things was apparently easier while taking the competence development course. During the course, the healthcare professionals had opportunities to discuss their use of the tools and how they handled the families with both experts and likeminded healthcare professionals. For some, it served as a safe space in which to develop their own teaching methods.

Finally, several healthcare professionals mentioned how having general experience with using tools as a method was important to improving the ease of implementing the Family Toolbox:

*Well, I also think that it’s because I’ve worked with health educational tools for many years, that I’ve felt ready, because if you’re new at this, it could be hard after only one day (of training). I don’t know how the others have felt it, but yes, you build on something that you’ve experienced from the other ones (toolboxes). Many of the tools you need to adjust a little, and I think that’s easier when you have some experience. (Interview 10).*

As mentioned, the physical therapist believed that, when implementing new tools, the chance of success is greater when one can build on earlier experience.

3) Introducing something new requires a significant commitment from the organization and management

When implementing the innovation, it was not only the healthcare professional’s planning and facilitating the course and the new tools that were important. Having colleagues and managers who support the course as well as an organizational and physical setting that was ready for the new course was also necessary:

*Well, I think it was really good, but it’s like being introduced to something that seems really exciting, but that’s not adapted to our everyday work. It’s also interesting I think, a really great idea, but we just can’t use it because we don’t see the whole family. (Interview 4).*

Most healthcare professionals mentioned how involving families could be difficult in terms of getting sufficient space, resources and manager support. Even though the educator believed family involvement was a great idea, it sometimes was simply not possible given the present conditions.

Another healthcare professional elaborates:

*There are good things in it, but how should I put it, there may be a limit in terms of resources. There will soon be an expansion of our effort to involve families, so I think doing that requires management support … we have four families attending on Monday, but that is out of a lot more families, and if you wanted to reach out to all the families… Then you’d need more time and that time would be taken away from something else. So of course we need management to support it and say that this is something that needs to be prioritized. (Interview 12).*

No matter how interesting and meaningful family involvement was to the healthcare professionals, manager support, time and resources were needed to implement the innovation fully. For all participants in the present interview study, the innovation inspired them and convinced them that working with family involvement could improve patient outcomes. As shown above, however, full success with implementation was not always enough.

Others were open to the chance of working with the Family Toolbox:

*Yes, I have a very good workplace. If you come up with new initiatives, they are very welcome. Of course you need to argue for it, but there’s a green light. Absolutely. Management is really excited about it, absolutely. And like I told you, it’s a pilot in terms of being something we can include in other courses too. (Interview 2).*

The same nurse elaborated further:

*Well, I think it’s been enormously inspiring, and it’s always about riding a positive wave that makes you want more. That’s how this is. It (The Family Toolbox) has come to stay, I think. Time will tell how and how far it will spread, but I really feel like I want to continue working with it. (Interview 2).*

The healthcare professional quoted above had a positive attitude toward implementing the innovation. Even though there was as yet no decision from the manager, there was a belief in the approach to involving families, and the healthcare professional seemed to have decided to keep working with the tools.

## Discussion

The healthcare professionals had generally positive attitudes toward implementing the Family Toolbox, and they could easily recognize that the themes described in the earlier needs assessment were important to patients who participated in the patient education sessions [9, 10]. The implementation process, including the competence development course and time for testing the tools and methods, was generally well received by the healthcare professionals, all of whom were committed to testing the toolbox during the 6-week course.

The existing literature highlights the importance being aware of the complexity of the implementation process when studying it [30]. This complexity is dependent on many factors, such as the context, setting, resources and implementation strategy [12, 13, 21]. This is confirmed by our study data, which are generally in line with the existing literature.

Like one of the categories of implementation research described by Nilsen [15], the present study was designed to examine the factors influencing implementation outcomes. For that reason, our analysis focused on important themes that could help healthcare professionals implement innovations in daily practice. Building on established knowledge from implementation science [11, 14], our main objective was to describe these themes as clearly as possible for both researchers and healthcare professionals to use.

The healthcare professionals in our study generally had positive attitudes toward the innovation and were excited to work with the tools and methods. The analysis revealed that focusing on the broader family, instead of the person with diabetes alone, inspired the healthcare professionals. This is very much in line with the study by Scaccia et al., which showed that organizational readiness partially comes down to motivation to implement an innovation [22]. In our study, the healthcare professionals described how involving the family members changed the patient education dialogue significantly – and that this motivated the professionals further.

Involving the families in patient education was associated with several barriers. First, facilitating larger and more complex groups was intimidating, and some healthcare professionals felt it involved too big a change. This was also referred to by Scaccia et al. as the innovation-specific capacities needed when implementing the innovation. In the present study, all participants mentioned this concern, but there were great differences in how they handled it. Some of them had less positive experiences and found it difficult to continue the intervention, while others perceived it as challenging, but exciting and as having great benefits. In the dataset, there was a tendency toward the healthcare professionals with the most general experience of facilitating education and with the most tool-specific experience also being the ones who searched for solutions and who kept trying to succeed. Moreover, these were the individuals who experienced the greatest benefits for the families.

The analysis showed that management, colleagues, resources and the setting available for education sessions had a great impact on the healthcare professional’s ability to believe in the implementation process and the sustainability of family involvement. Several implementation studies have suggested that the context plays a very central role in the chance of having a successful implementation process [20, 21]. Again, this is in good accordance with the findings of Scaccia et al. and the last part of organizational readiness – the general capacity of the organization. Furthermore, it highlights the need for organizational readiness and the ability to implement an innovation while handling other more operational tasks at the same time. In the present study, all of the municipalities were implementing the Family Toolbox while running a pre-planned patient-oriented patient education program. In the analysis, this acted as a case-specific barrier.

The many existing barriers affecting the implementation process revealed the importance of adapting future implementation studies to the context [26]. In the present study, the Family Toolbox had been developed as a very flexible tool that can be adapted to the context in terms of room size, number of participants, number of healthcare professionals, theme in focus, etc. The implementation process was designed to encompass all of the different municipalities with different profiles, but in practice not all of the healthcare professionals managed to make room for the innovation. Again, this calls for viewing implementation as a highly complex matter and as an independent research area of healthcare practice that needs to be studied further.

The findings of this study are valuable for the further implementation of The Family Toolbox, which is made freely available to all municipalities in Denmark along with the detailed guide on family involvement. Likewise, The Capital Region of Denmark and The Region of Southern Denmark will continue to offer competence development to healthcare professionals interested in involving family members in patient education. This mix of educational guidance, written guidance and explicit tools provides the healthcare professionals with several options when acquiring knowledge and skills on family involvement in patient education. This is important to meet the complex needs of implementation [16, 17, 20, 21].

The present study could raise the question on whether family members should more often be involved in the care and rehabilitation of patients throughout the healthcare system. Both in the literature [1, 2] and in this study, there seems to be immediate benefits, but these benefits should be measured against potential harms and the expected costs. There is a need for future research on how family involvement on a bigger scale would affect our healthcare system and how healthcare policy could encourage the change.

Working with a tangible, well-described and tested innovation made the interviews focused and easy to conduct. All of the interviews were therefore concrete and relevant to the analysis. Another strength was that working with an engaged group of healthcare professionals enabled us to choose a diverse and well-suited group for the interviews.

On the contrary, this could also limit the study, as the professionals included in the interviews are all professionally interested in family involvement, as they partook in the Family toolbox course [27]. This could cause for selection bias, which we were aware of during the interviews and the subsequent analysis. Performing the interviews shortly after the professionals’ training course is another limit, as it might not be possible to illuminate the entire implementation process after such a short period of time. On the other hand, the process of implementing the innovation was easily remembered by the healthcare professionals, presumably allowing them to elaborate in more detail. The present study, however, only reflected the first phase of implementing the tools. Furthermore, it is solely the experience of the healthcare professionals and their view on the implementation process that were in focus. In future research, it will be necessary to engage with municipal management and gather their views on the implementation process as well.

## Conclusions

The study shows that it is feasible to implement an innovation to involve family members and close relatives in complex patient education sessions in Danish municipalities. It is, however, highly dependent on the commitment of the healthcare professionals delivering the education and the manager responsible. Facilitating the bigger and more complex groups of people including family members requires great openness from healthcare professionals but contains potential benefits for everyone involved. The thematic focus on families seems to be a great inspiration to healthcare professionals.

## Data Availability

As study data is in Danish it has not been submitted along with the article.
